# Recent Advances in Chemical Sensors Using Porphyrin-Carbon Nanostructure Hybrid Materials

**DOI:** 10.3390/nano11040997

**Published:** 2021-04-13

**Authors:** Gabriele Magna, Federica Mandoj, Manuela Stefanelli, Giuseppe Pomarico, Donato Monti, Corrado Di Natale, Roberto Paolesse, Sara Nardis

**Affiliations:** 1 Dipartimento di Scienze e Tecnologie Chimiche, Università di Roma Tor Vergata, Via della Ricerca Scientifica, 1, 00133 Rome, Italy; gabriele.magna@uniroma2.it (G.M.); federica.mandoj@uniroma2.it (F.M.); roberto.paolesse@uniroma2.it (R.P.); nardis@scienze.uniroma2.it (S.N.); 2 Dipartimento di Medicina Molecolare e Traslazionale, Università di Brescia, Viale Europa 11, 25123 Brescia, Italy; giuseppe.pomarico@unibs.it; 3 CSGI, Research Center for Colloids and Nanoscience, Via della Lastruccia 3, 50019 Florence, Italy; 4 Dipartimento di Chimica, Università La Sapienza, piazzale Aldo Moro 5, 00185 Rome, Italy; donato.monti@uniroma1.it; 5 Dipartimento di Ingegneria Elettronica, Università di Roma Tor Vergata, Viale del Politecnico 1, 00133 Rome, Italy; dinatale@ing.uniroma2.it

**Keywords:** porphyrins, carbon materials, nanostructures, hybrid materials, graphene, carbon nanotubes, carbon dots, chemical sensors

## Abstract

Porphyrins and carbon nanomaterials are among the most widely investigated and applied compounds, both offering multiple options to modulate their optical, electronic and magnetic properties by easy and well-established synthetic manipulations. Individually, they play a leading role in the development of efficient and robust chemical sensors, where they detect a plethora of analytes of practical relevance. But even more interesting, the merging of the peculiar features of these single components into hybrid nanostructures results in novel materials with amplified sensing properties exploitable in different application fields, covering the areas of health, food, environment and so on. In this contribution, we focused on recent examples reported in literature illustrating the integration of different carbon materials (i.e., graphene, nanotubes and carbon dots) and (metallo)porphyrins in heterostructures exploited in chemical sensors operating in liquid as well as gaseous phase, with particular focus on research performed in the last four years.

## 1. Introduction

**Figure 1:**
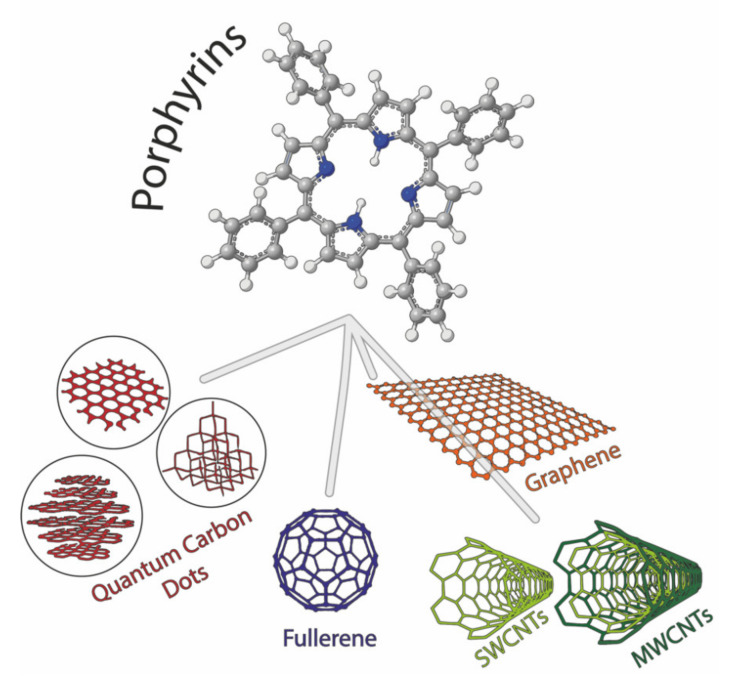
Porphyrin macrocycle (utilizing 5,10,15,20-tetraphenylporphyrin (TPP) structure as exemplificative backbone) and the different carbon material structures discussed in the present work.

Chemical sensors research and development have been engaged for so long in fabricating simple and reliable devices able to perform rapid and on-site detection of chemical species in order to readily face potential emergency scenarios dealing with safety, environment and health applications, where speed of response can make a real difference [1,2,3,4,5,6,7,8,9]. Given the extreme variability in nature as well as critical amount of target analytes, ongoing efforts are focused to improve both sensing materials and transducers performances, along with chemometric techniques that are fundamental for data sets with many variables, i.e., complex/real matrices. Chemical receptors can be virtually selected among inorganic and organic materials, although this latter category lends itself to undergo straightforward and creative modifications designed ad hoc for a specific purpose. Among them, porphyrins are undoubtedly exploited in a large number of systems used in chemical sensing [10,11,12], the main reason being the peculiar optical, catalytic and electrochemical properties, easily tunable by well-established synthetic protocols, which enable their use in different transduction modes. Similarly, carbon nanomaterials are extensively investigated and applied in chemical sensors [13,14,15,16,17], thanks to their distinctive intrinsic optical, electronic and magnetic properties, biocompatibility, chemical versatility and absorption capability. Despite their remarkable achievements as individual elements, their mutual combination in hybrid nanostructured materials often revealed a winning strategy, leading to increased performances and novel opportunities to transduce the porphyrin-analyte interaction in a measurable signal [10,12,18,19]. This topic is abundantly covered in literature, and we have highlighted here the recent examples reported in the last four years, almost narrowing the field of carbon nanomaterials (CNMs) to graphene, carbon nanotubes and dots (Figure 1).

## 2. Graphene@Porphyrin Sensors

Graphene is probably the most combined CNM with porphyrins for sensing applications. This CNM is a planar 2D material consisting of sp^2^ carbon atoms linked via covalent bonds, which can further organize in thick multi layered graphitic nanosheets. It is primarily obtained by mechanical and liquid phase exfoliation of graphite and, by chemically treating of the surface, it is possible to generate different forms as photoluminescent graphene oxide (GO) and reduced graphene oxide (rGO), equally useful in chemical sensors. Electrons flowing through sideways overlapped unhybridized p-orbitals confers the aromatic character, accounting for its outstanding conducting properties. Additionally, a multitude of superior characteristics in term of high surface-to-volume ratio, carrier mobility, thermal conductivity, optical transmittance and mechanical flexibility warrant its beneficial use for sensors [20]. Several routes have been followed to combine this excellent material with equally valuable (metallo)porphyrins [21], producing novel hybrid materials with improved features that lend themselves to be integrated with different transducers. Below, a brief survey of recent examples is given, reported in literature bundled according to the transduction mode involved (i.e., optical, electrochemical/photoelectrochemical, conductometric).

### 2.1. Optical Sensors

**Figure 2:**
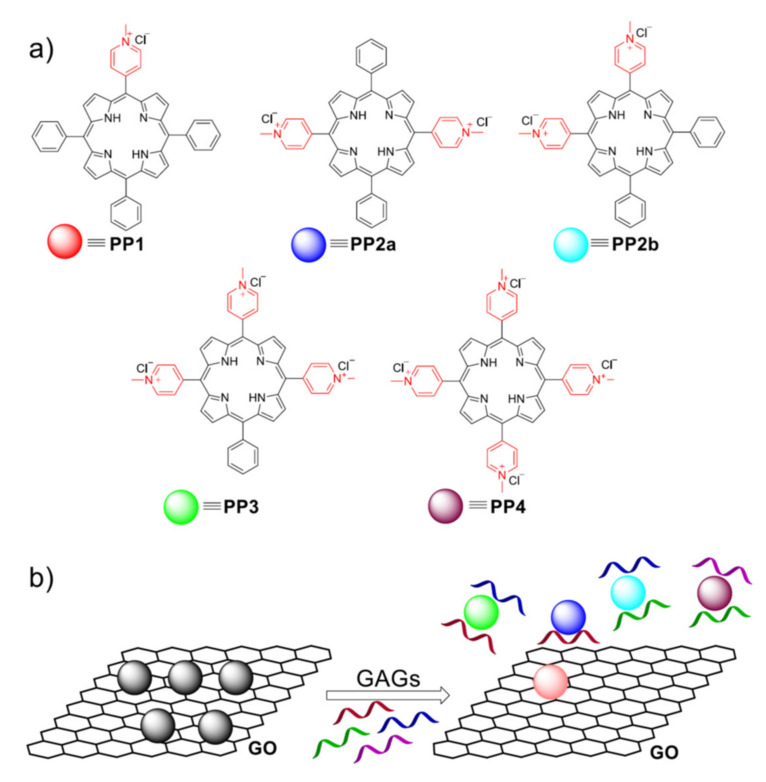
(**a**) Molecular structures of the positively charged porphyrins and (**b**) the working principle of the sensor arrays developed by Zhang and coworkers. Adapted with permission from [22]. Copyright 2020, American Chemical Society.

In optical chemical sensors, the target analyte is detected by quantifying the changes of one of the optical properties of the sensitive material, typically absorbance or luminescence. Porphyrins feature intense and distinctive absorption and emission bands, enabling the straightforward use even with consumer electronics set-up such as combination of LED, photodiode and optical filter. Zhang and coworkers reported the use of nanocomposites, obtained by assembling a set of positively charged porphyrins (Figure 2a) onto graphene oxide, to construct a NIR fluorescent sensor array for discrimination of Hep from its glycosaminoglycan (GAG) analogues [22].

Hep is in fact largely used in clinics, both as prophylactic and therapeutic drugs: It is not artificially prepared, but isolated from animal tissue, together with other GAGs, so its discrimination is crucial. Five nanocomposite materials were synthesized by mixing different porphyrins (Figure 2a) and the fluorescence quencher, GO, in a (phosphate-buffered saline (PBS) buffer solution: each of them was placed into a single row of a well-plate, generating a 5 sensors array. In presence of GAGs, the positively charged porphyrin is removed from the GO surface through the binding with the analyte and its fluorescence recovered (Figure 2b). The emission intensities of each well were measured after the addition of 4 different GAGs, including Hep, and the obtained data matrix analyzed by hierarchical clustering analysis, to understand the driving force for the pattern recognition, while Linear Discriminant Analyses was applied to check the ability of the array to discriminate among different GAGs. The authors reported that the array was able to differentiate Hep from biologically abundant anions and biomolecules in various concentrations, suggesting its reliability for detection of this analyte in biological samples.

A highly fluorescent sensor for copper ions, which has a vital role in several biological processes, has been realized using hematoporphyrin (HP) functionalized graphene oxide nanosheets, taking advantage of the high affinity of the pyrrolic macrocycle for Cu^2+^ [23]. The copper ion induces the quenching of the fluorescence of the hybrid material, synthesized by esterification reaction in presence of sulfuric acid, at different concentrations in water. The authors have also evaluated the effect of pH and that of 15 interfering metal ions on the fluorescence intensity; according to their results, the sensor has a detection limit of 54 nM, thus could be used in real water sample.

**Figure 3:**
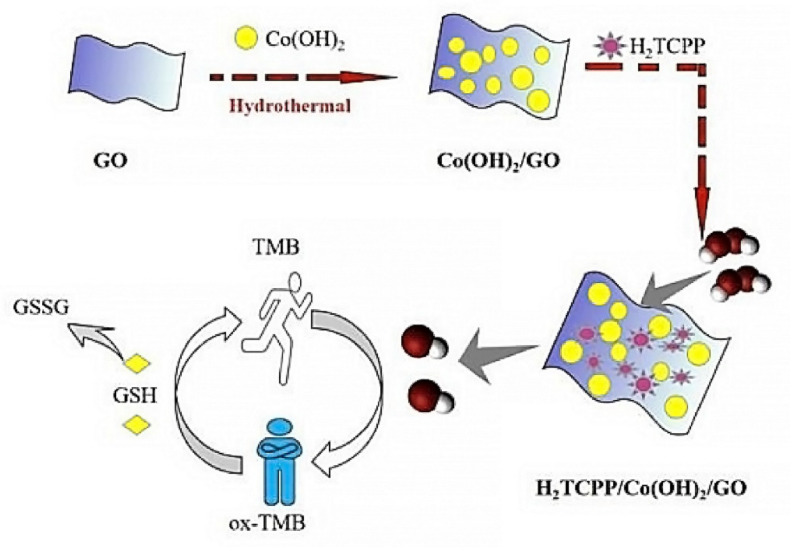
(TCPP)/Co(OH)_2_/graphene oxide (GO) fabrication route. In presence of H_2_O_2_, hydroxyl radicals are produced that in turn oxidize the 3,5,5-tetramethylbenzidine (TMB). oxTMB is now capable of sensing glutathione (GSH) by a reducing process. Adapted with permission from [24]. Copyright 2019, Royal Society of Chemistry.

Catalytic or photocatalytic behavior of porphyrins has been largely exploited in chemical sensors, for example to activate/accelerate reaction chains that ultimately activate or deactivate a dye indicator (on/off mechanism). Recently, Xin Zhao et al. have found that a ternary system composed by modified Co(OH)_2_ deposited on the surface of GO and the 5,10,15,20-tetrakis(4-carboxyphenyl)porphyrin (TCPP) has enhanced peroxidase-like activity and is able to catalyze the oxidation of 3,5,5-tetramethylbenzidine (TMB) by hydrogen peroxide [24]. In this work, TCPP/Co(OH)_2_/GO ternary system demonstrated good dispersibility as well as an excellent peroxidase-like catalytic activity (pH = 4 and T = 25 °C as optimal conditions), which is peculiar of the composite, since it was not found in the absence of one of the three components (Figure 3). Concerning photocatalytic mechanism, visible irradiation produces photoexcited electrons in porphyrins, which in turn can be transferred to Co(OH)_2_ obtaining charge separation at the interface. Charge separation phenomena are very common features of hybrid interfaces when the charge transfer mechanism hinders the recombination of photoinduced electron–hole pairs. In the TCPP/Co(OH)_2_/GO material, separated charge carriers tend to form reactive species that can efficiently reduce the H_2_O_2_ adsorbed onto the composite surface into H_2_O and reactive OH^•^ species. Finally, these catalytic properties were utilized to activate, by oxidation, the TMB that in turn served as glutathione (GSH) colorimetric sensor. ox-TMB is reduced when interacting with GSH, turning back from blue to colorless. This mechanism allows for the quantitative detection of GSH in the range of 1.5–500 μM, with a limit of detection (LOD) of 9.5 μM. Selectivity was investigated by measuring 10 time more concentrated solutions of some amino acids, saccharides and metal ions, proving the superior sensing properties to GSH with respect to interfering substances such as fructose, glucose, maltose, Cu^2+^, Cd^2+^, Ni^+^, Fe^3+^, Trp, D-serine, and uric acid.

### 2.2. Electrochemical and Photoelectrochemical Sensors

Electrochemical sensors are typically used for the detection of targeted compounds in liquid phase, thanks to the direct measurement of an electrical signal related to the analyte concentration, which results from an electrochemical process occurring at the electrode−solution surface. Electrochemical sensors can be divided in two categories, namely, potentiometric and volt-amperometric techniques, where the electrode potential at zero current flow and the current flowing in the cell resulting from redox electrodic reactions are measured, respectively. Several examples belonging to this category have been recently reported in the literature.

**Figure 4:**
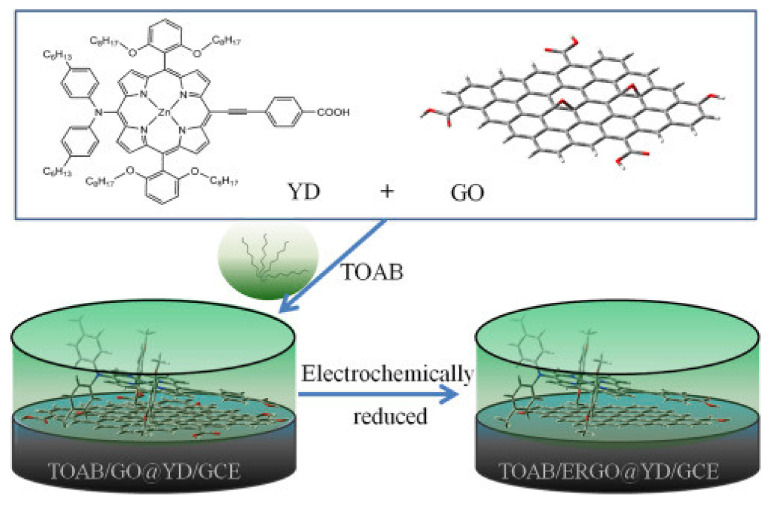
Nanohybrid system developed by Fan and co-workers for ascorbic acid sensing. Adapted with permission from [25]. Copyright 2018, Elsevier.

A nanohybrid based on zinc porphyrin noncovalently functionalized graphene oxide has been obtained by Fan and co-authors by hydrogen bonding and π–π stacking interaction and applied as non-enzymatic electrochemical sensor [25]. The scarce solubility of the employed porphyrin limited its application in electrochemistry, but the authors enhanced the inner electroactivity of the macrocycle by addition of a cationic surfactant (TOAB) and the combination with electrochemically rGO: Such a strategy improved the electron transfer between porphyrin and the electrode (Figure 4). The fabricated system has been studied for ascorbic acid sensing also in real samples, showing high sensitivity (13.58 mA mM^−1^), a wide linear concentration from 1.33 mM to 1.46 mM (R = 0.998), and a limit of detection as low as 0.28 mM.

**Figure 5:**
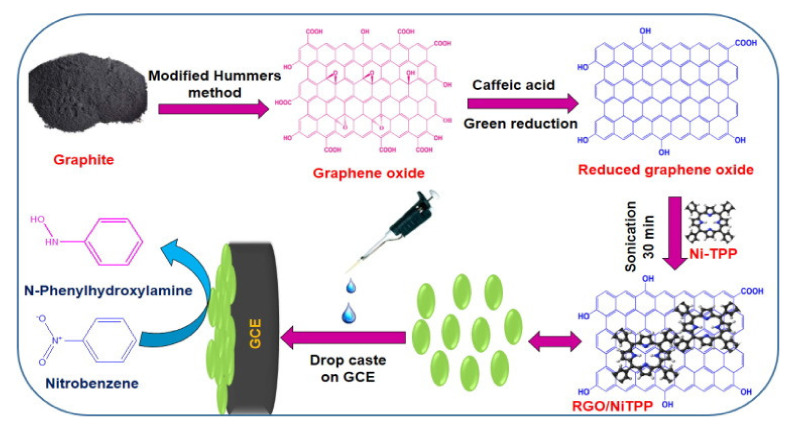
Chemical reduced graphene oxide (CrGO)/NiTPP/GCE modified electrode for the detection of nitrobenzene. Adapted with permission from [26]. Copyright 2017, Elsevier.

An environmentally friendly method has been applied by Kubendhiran and co-authors [26] for the green preparation of a GrGO/Ni-TPP composite, utilizing caffeinic acid as the reducing agent for GO and taking then advantage of the π-π stacking interactions between the GrGO obtained and the porphyrin complex. The obtained nanocomposite has been deposited by dropcasting on a glassy carbon electrode giving rise to a sensor for the determination of nitrobenzene (Figure 5), a carcinogenic organic compound used for the production of explosives, herbicides, insecticides and dyes whose determination has become a priority due to its tendency to accumulate in the environment. Such an electrode performances have been compared to bare GCE, GrGO, NiTPP and GO/NiTPP by CV analysis, obtaining for the GrGO/NiTPP the highest reduction peak current towards the reduction of NB to N-phenyl hydroxylamine; sensitivity, linearity and limit of detection, instead, have been studied by the more sensitive DPV technique. GrGO/NiTPP sensor demonstrated a good selectivity for NB even in presence of common interfering molecules and ions, making it suitable for practical applications in industrial samples.

A new tetraruthenated porphyrin species containing a (CoTPyP) connected to four [Ru(H_2_dcbpy)_2_Cl]^+^ complexes, has been synthesized and successfully deposited onto GO sheets [27]. Nickel (II) ions were added to overcome the effect of the high negative charge of the deprotonated complex, limiting the oxidizing catalytic properties of the Co(III), due the electrostatic repulsion with the carboxylate groups of the GO surface. In this way, a stable [CoTRP(dcbpy)_2_]-Ni/GO layered material was in situ generated and fully characterized. The GC electrodes modified with the composite were used in BIA for the determination of the antibiotic isoniazid (pyridine-4-carboxilyc acid hydrazide): The amperometric sensor proved capable of working under very mild conditions, with a detection limit of 3.5 μM.

rGO, due to its favorable electron mobility and unique surface properties, can accommodate active species and facilitate their ET at electrode surfaces, promoting for instance the coordination reaction between metal ions and porphyrin derivatives. Li and collaborators proposed an electrochemical sensor for cadmium ions detection in water samples, enhancing the rGO-porphyrin nanoconjugate-based features with AuNPs [28]. Gold nanoparticles, whose aggregation is prevented by the use of rGO as supporting material, can enhance ET between the redox centers of biomolecules and electrode surfaces and can also act as catalysts to increase the rates of electrochemical reactions, resulting in an enhanced sensitivity. The sensor also revealed a good selectivity: No clear responses have been obtained upon the addition of other metal ions including Fe^3+^, Na^+^, Mg^2+^, Ca^2+^, and Mn^2+^ except for Hg^2+^, which having a different reduction peak position respect to that of Cd^2+^ does not interfere with the electrochemical detection of cadmium ions.

The simultaneous detection of HQ with CC was reported by Liu and co-workers using an electrochemical sensor based on poly(MTFPP)/GO hybrids (M = H_2_, Co and Zn) on GCE, with the best results obtained in the case of cobalt-containing hybrid [29]. The two analytes were concurrently quantified through the good current peaks separation, achievable thanks to the synergistic effects of GO and CoTFPP that increased and made faster the electron transfer process towards the two molecular targets. Sensitivity and LOD values were 10.40 μA∙μM^−1^∙cm^−2^ and 0.17 μM for CC, and 8.40 μA∙μM^−1^∙cm^−2^ and 0.21 μM for HQ.

A copper porphyrin salt (Na_4_CuTCPP) has been used as intercalator in the liquid-phase non-covalent exfoliation of GSs [30]. The functionalized nanosheet has been used to modify a GCE for the simultaneous voltametric determination of acetaminophen and dopamine, both in pharmaceutical preparations and in human serum.

The performances of the Na_4_CuTCPP/graphene electrode were compared to those of copper free Na_4_TCPP/graphene and graphene exfoliated in pure DMF: The study demonstrated that the metal complex not only assists the exfoliation process but also acts as an enhancer to improve the electrochemical oxidation of the two analytes on the modified electrode, due to the large specific area of graphene and the electrocatalytic properties of the porphyrin complex.

Besides the potentiometric and volt-amperometric methods, we can also include the photoelectrochemical one, which is a rather new technology where active species are excited by light and transfer the electrons to electrode, giving the current signal. In this case, the separation between the irradiation source and the electrochemical detection assures higher sensitivity and specificity if compared with both optical and electrochemical methods. The exploitation of porphyrins as photosensitizers to enhance photocurrent intensity is related to their photoelectronic properties, like their wide photoresponse range in visible and near-infrared regions and their quick time of the charge of recombination between the HOMO orbital and the hole of oxidized porphyrin. As an example, we report here the work of Lu and co-workers that used rGO, obtained by reduction of GO with CS, and added AuNPs to improve the conductivity of nanocomposites [31]. Water-soluble Zn- and CuTCPP complexes were added by drop casting technique, resulting in the formation of a π–π nanoassembly. The nanocomposites, deposited on ITO electrodes, have been then characterized: the best performing system, rGO/AuNPs/CS/ZnTCPP was used as “on and off” PEC sensor for the determination of HQ, a toxic organic compound, whose presence greatly increases the photocurrent response. According to the proposed mechanism, the photoexcited electrons of ZnTCPP were injected into the rGO and then transferred to AuNPs and further to the ITO. When HQ was introduced into the system, it acted as sacrificial electron donor providing electrons to metalloporphyrin molecule, decreasing the percentage of recombination and enhancing the photocurrent signal. The increase in photocurrent was attributed to the oxidation of HQ to BQ that boosts the efficiency of the charge separation. A similar approach has been used by the same group for 4-NP detection using nanocomposite consisting of GO-CO_2_H functionalized with ZnAPTPP through π–π interactions, loaded with AuNPs [32]. In another work they reported the use of a one-dimensional TCPP/NS-GO composite, in presence of K_2_S_2_O_8_ as co-reactant, for the ECL detection of Fe^3+^ in human serum, finding a greatly enhanced ECL intensity compared to TCPP NS/ K_2_S_2_O_8_ or TCPP/NS-GO [33]. According to the authors, the coexisting dissolved O_2_ into the buffer played a key role in the ECL increase, since it accelerated the decomposition of K_2_S_2_O_8_. The high ECL intensity was strongly quenched by iron (III) species that were optimally bound by the surface functional groups (hydroxyl and carboxyl) of the composite, as indicated by FT-IR and UV–VIS spectroscopies.

### 2.3. Other Application for Graphene-Porphyrin Hybrids

In this section, we present just few examples using graphene-porphyrin hybrids in biosensors and field-effect transistor (FET) devices with potential applications in biological field.

A nanocomposite material consisting of CrGO and tetraphenylporphyrin derivatives, bearing one or four butyloxyphenyl groups, was prepared and fully characterized by Korri-Youssoufi’s group [34]. The presence of these peripheral groups on the porphyrin skeleton improved both the dispersibility and the electron transfer ability. Furthermore, the non-covalent functionalization of CrGO, obtained through π–π interactions in the case of the mono-substituted porphyrin and additional hydrogen bonds for the tetrasubstituted macrocycle, led to nanocomposites suitable for bioconjugation, thus useful for the fabrication of biosensors devices. The authors reported the use of tetrabutyloxy-substituted porphyrin/CrGO hybrid for the detection of DNA, with a LOD of 1 attomolar and evidenced the ability of the system to discriminate between DNA from Rpob gene of M. Tuberculosis strand and mutated in PCR sample.

**Figure 6:**
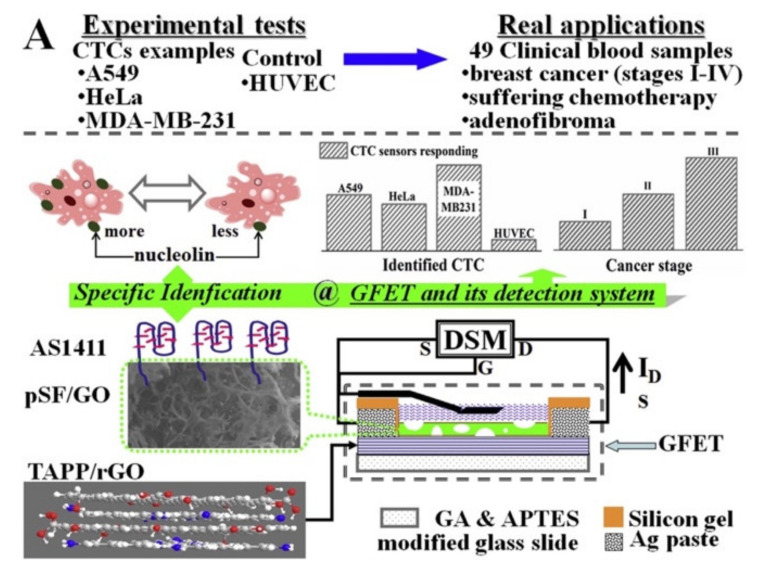
Experimental set-up to identify circulating tumor cell (CTC) by means of porous silk-fibroin (PSF)/GO-5,10,15,20-tetrakis(4-aminophenyl)porphyrin (TAPP)/rGO-field-effect transistor (FET). Adapted with permission from [35]. Copyright 2019, Elsevier.

One more example, given by Jia and coworkers, reported a porphyrin mediated rGO field effect transistor for circulating tumor cell (CTC) assay (Figure 6) [35]. 5,10,15,20-tetrakis(4-aminophenyl)porphyrin (TAPP)/rGO was prepared by using Hummer’s method to get GO, followed, after dispersing the mixture GO:TAPP (4:1) in deionized water, by hydrazine-ammonia reduction. The hybrid system was then used as channel material. The sensing interface was established on the channel surface of TAPP/rGO-FET which was decorated by porous silk-fibroin (PSF)/GO, able to accommodate cells. The presence of hydrogen evolution reaction taking place on this hybrid material enhanced charge transfer improving the original rGO conductivity. AS1411 (a classical CTC aptamer) was used to functionalize the above mentioned PSF/GO-TAPP/rGO-FET, because of its specificity to the over-expressed nucleolin sites on CTCs. The AS1411 captured CTCs can be identified by the currents responding of graphene FET. Human lung cancer cell line A549, breast cancer MDA-MB-231 and cervical cancer HeLa control, indicating the PSF/GO-TAPP/rGO-FET based CTC sensor is a promising candidate for clinical assay.

## 3. Carbon Nanotubes@Porphyrin Sensors

CNTs are monodimensional tubular nanostructures consisting of single or multiple rolled-up graphene sheets, named SWCNTs and MWCNTs, respectively [36]. They possess a different diameter length, being less than 1 nm for the former and reaching more than 100 nm for the latter. If their unique thermal and mechanical properties account for their wide investigation in material science, their electrical features make them outstanding candidates for many applications, comprising sensors and transistors development. In particular, MWCNTs are always conducting metal-like materials, while SWCNTs’ conductivity spans from metallic to semi-conducting or insulating, depending on the rolling-up direction of the graphene layers, i.e., the chiral vector. Based on these properties, these materials are especially applied in electrochemical sensors and FET devices for the detection of analytes in liquid and gaseous phases, respectively. The combination with porphyrins resulted extremely beneficial even for these CNMs, as discussed hereafter.

A sensing platform to detect cortisol in saliva and suitable for point of care diagnosis has been reported by Bhansali’s group, consisting of SPCE electrodes covered by the MTPP/MWCNTs hybrids [37]. Novelty of the work was the use of metalloporphyrin catalysts for the detection of the target analyte in alternative to antibodies or aptamers traditionally employed for the scope. The strong π-stacking interaction within the stable composite material enhances the electronic and optical properties of porphyrins, as well as their electrochemical (i.e., fast electron transfer) and catalytic activities. Among the three metalloporphyrins tested (Co, Ni and Cu, respectively), CuTPP was the best in cortisol electrocatalytic detection, as also indicated by computational studies giving the higher binding energy for cortisol (−15.15 kcal/mol) with this complex. CuTPP/MWCNTs electrodes displayed excellent current response to increasing cortisol concentrations within the range of 50 fM–100 nM. To validate the proposed analytical method, the detection of salivary cortisol levels in young adult women have been performed by either the fabricated sensor or the standard ELISA method. Results of the two methods were in good agreement, even if the achieved analyte concentrations were slightly different, possibly because of electrochemical interferences from saliva.

A universal miniaturized and portable electrochemical sensing platform for analysis of Zn^2+^ levels in samples of varied nature has been proposed by Mondal and Subramaniam [38]. They fabricated the platform assembling two coaxial, cable-type electrochemical sensors consisting of a bare CNT-thread (acting as counter/reference electrode) and the analogous uniformly covered by the porphyrin ionomer membrane (TAPP), attaching them to conductive copper tape and sandwiching between two laminated PET substrates. The fabrication method resulted very easy and cost-effective and afforded a system with dimension comparable to that of a two-rupee coin. The proposed sensing platform resulted highly selective for Zn^2+^ (selectivity coefficient 10^−3^−10^−5^) respect to several metal cations (Na^+^, K^+^, Mg^2+^, Cd^2+^, Ca^2+^, Fe^2+^, and Cu^2+^) and anions (Cl^−^, NO_3_^−^, PO_4_^3−^, and CH_3_CO_2_^−^) and enabled the rapid (<1 min) real-time detection of Zn^2+^ in human perspiration and agricultural soil samples, thus with important applicative impact in medical diagnosis and soil-nutrient assessment fields, respectively. Notably, the system was successfully used to detect the target molecule through different electrochemical techniques, as cyclic voltammetry, differential pulse voltammetry and chronoamperometry, over a wide dynamic range (0.1–500 ppm) and pH spanning from 4.5 to 7.0, showing suitable for the analysis of samples of very different compositions and nature.

Concerning the detection of analytes in the vapor phase, CNTs operate nearly always in conductometric devices based on interdigitated electrodes or FET. From their part, metalloporphyrins may be layered on nanotubes by simple drop casting, due to the electrostatical interaction between the two large conjugated systems possessed by both porphyrins and SWCNTs. Alternatively, pyridine groups may be immobilized on CNTs to offer coordinative sites to porphyrin metal complexes. In this latter case, the functional groups are sparse onto the surface, around 1.4 per 100 SWCNT units.

In two recent manuscripts [39,40], Pauly, Ndiaye et al. investigated the effect of peripheral substituent groups on the uptake of BTX vapors. BTX are excellently sensed by porphyrin/CNTs systems because of their aromaticity (π–π interactions). The authors utilized QMBs and conductometric methods to investigate the mutual effects of macrocycles peripheral groups (*tert*-butyl, phenyl, ethyl) and volatile vapors of BTX (methyl group). Initially only the macrocycles were deposited onto QMBs and, as expected in this case, the nature of the peripheral moieties (aryl or alkyl) dictates the kinetic of time response of both adsorption and desorption events. On the other hand, in case of hybrid material, the functionalization only partially covers the CNTs surface leaving naked nanotubes surface exposed to environment. In account of this, the authors suggest to consider the presence of two different adsorption sites: one for porphyrin and one for bare CNTs. When the QMB experiment was replicated considering hybrid materials, methyl groups of the BTX result the dominant parameter that modulates the response profile whatever the peripheral substituent of hybrid materials: the π-alkyl interactions reinforce the π–π interactions resulting in stronger bindings. With the increasing of alkyl groups (where styrene > toluene > benzene), the desorption curves present a slow and more persistent desorption. Finally, the influence of organic coating is even less evident in case of conductometric measurements since porphyrins do not contribute to the overall resistance (due to the quasi-insulating nature of materials). Under this condition, even if the gaseous molecules adsorbed on the functional moieties, the contribution to the electrical conduction will be negligible. This result is expected since in this case porphyrins cannot inject electrons into CNTs and BTX interacts only by electrostatic forces. Thus, the main contribute of porphyrins is supposed to be the capability to catalyze redox reactions.

Furthermore, Rushi et al. studied the influence of post functionalization annealing in tuning the sensing properties of FeTPP functionalized/SWCNTs to benzene [41]. CO_2_H-SWCNTs were dielectrophoretically aligned to form framework that was non-covalently functionalized by FeTPP. Authors investigated the effect of three annealing temperatures (45, 90 and 150 °C) on the hybrid material. Annealing produced a resistance decrease along with a reduction of average diameter of functionalized nanotubes. The main benefit of annealing (at 90 °C) appeared to be a small drift of baseline over the time and a faster recovery from benzene exposure; these benefits were obtained in exchange of smaller intensities of response.

A further example was reported by Hui Wang and coworkers that utilized different metalloporphyrins to produce an array based on six FET sensors where source and drain were connected by hybrid CNTs to detect volatile organic compounds (VOCs) helpful to diagnose Huanglongbing, a bacterial disease of citrus, at the asymptomatic stage [42]. The study case was focused on a set of VOCs (tetradecene, linalool, nonadecane, phenylacetaldehyde, and ethylhexanol) which were found to be correlated to the asymptomatic stage of Huanglongbing disease in plants. SWNTs were casted from solution onto single gap microelectrodes and further functionalized with metalloporphyrins MTPP (M = Zn, Cu, Fe, Cu) and MOEP (M = Cu and Mn) by solvent evaporation technique. Seven different concentrations were measured for each VOC and an artificial neural network model was trained to predict the chemical identity and the concentration of vapor samples. Results showed that the neural model outperformed PLSR obtained satisfactory capacity in accurately predict concentrations. In the case of FET devices, Swager et al. found that gate voltage may modulate the sensitivity of sensors based on F-SWCNTs and FeTPPClO_4_, which serves as redox active CO binding site [43]. UV–VIS spectroscopy, differential pulse voltammetry, and density functional theory reveal that the porphyrin is responsible for the in-situ reduction of Fe^III^ to Fe^II^, which in turn enhances the interaction between the F-SWCNTs and CO. As above mentioned, one of the main novelties of the work is the possibility to modulate (in this case amplifying) the sensitivity to CO when negative gate voltage is applied. Negative voltage modifies Fermi energy level promoting the in situ reduction of iron in porphyrin thereby increasing the affinity towards CO. Finally, concerning the density of pyridyl groups, optimal functionalization was found when 1.4 pyridyl are linked per 100 SWCNT units, balancing the degree of functionalization and preserving the SWCNT sp^2^ networks characteristic. The lowest detected concentration was 80 ppm of CO in N_2_, and humidity (RH = 41%) was proved to do not significatively influence the response.

**Figure 7:**
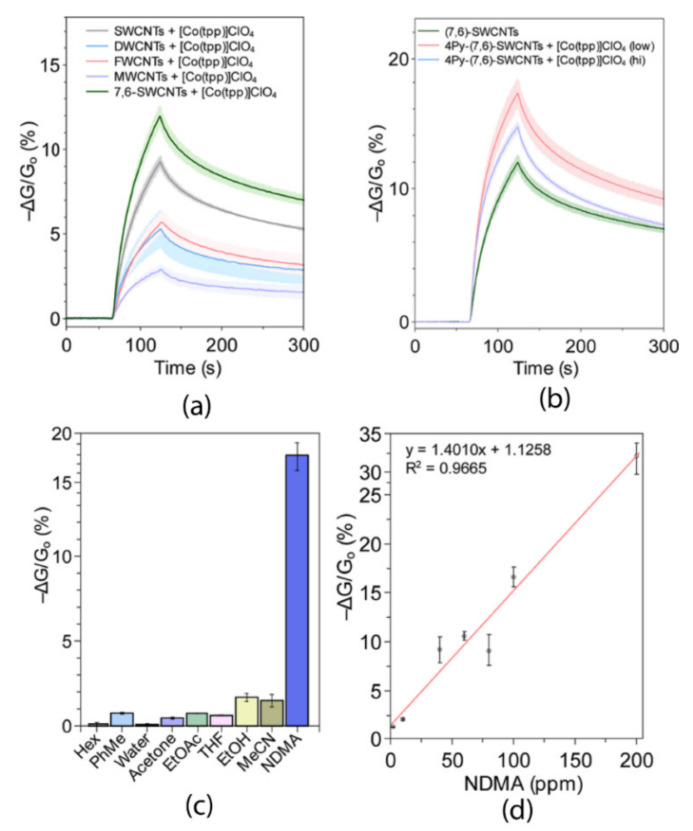
(**a**) Effect of different tubular structures (single, di-, few, and multi-walled nanotubes) on sensor response to *N*-nitrosodimethylamine (NDMA). (**b**) Effect of covalent porphyrin binding on nanotubes at different ratios (low and hi have 1.4 and 1.9 pyridyl groups per 100 carbons, respectively). (**c**) Sensor selectivity pattern to a set of volatile organic compounds (VOCs) utilized as chemical probes and (**d**) calibration curves at different concentrations. Adapted with permission from [44]. Copyright 2019. American Chemical Society.

By changing the coordinated metal, the same group reported in a second manuscript the superior properties of CoTPP/F-SWCNTs to sense *N*-nitrosodimethylamine (NDMA), when compared to both other carbon nanotube structures (single, double, few, and multi-walled nanotubes (Figure 7a)) and to materials functionalized by simple mixtures of cobalt(III) TPP and unfunctionalized CNTs (Figure 7b) [44]. In the case of cobalt metalloporphyrin, the exposure of the devices to VOCs containing different functional groups only showed very small responses (Figure 7c) confirming the possibility to utilize the sensor to detect ppm levels of NDMA. ^1^H NMR and UV–VIS spectroscopy confirmed the excellent propensity of Co-TPP to form complexes with NDMA. In addition, the authors optimized the device to detect ppb levels of NDMA by utilizing a relatively small channel gap that minimizes the layer thickness and allows for a more efficient vapor diffusion throughout the active sensing layer, leading to higher sensitivity (Figure 7d). Finally, the sensor results extremely sensitive to NDMA, NDEA, and NDBA (LOD = 1 ppb) and can be integrated to distributed environmental air monitoring.

A completely different approach to combine porphyrin to SWCNTs is proposed by Lee et al. that fabricated hybrid sensing material combining TiOTPyP nanofiber and SWCNT to detect hydrogen peroxide vapors [45]. Detection of H_2_O_2_ in the exhaled breath is important in clinical analysis because it can be correlated to several diseases. Authors produced nanofibers of a TiOTPyP by surfactant-assisted self-assembly method and mixed in solution with SWCNTs. Network of nanofibers and nanotubes was formed by spray casting onto the substrate. The choice of the porphyrin complex TiOTPyP was due to its capability to react with H_2_O_2_ forming the corresponding monoperoxo complex. Sensor performances were tested in the 0.1–10 ppm range of vapor pressure of H_2_O_2_ and compared with some interferents, showing weak response to NH_3_ gas and negligible response to other usual constituents of exhaled breath. Stability was tested over 100 days, proving that sensor is stable with less than 5% change in response during the period.

## 4. Carbon Dots@Porphyrin Sensors

CQDs often just carbon dots (CDs) are 0D quasi-spherical nanomaterials containing sp^3^ hybridized carbon atoms, whose diameter covers the 2–10 nm range. They can be prepared from a variety of carbon sources, including waste materials, by using electrochemical or hydrothermal methods, pyrolysis and microwave synthesis [46]. Fluorescence is surely the most attractive property of these materials, that recently have emerged as highly promising sensing materials and excellent agents for bioimaging and theranostic thanks to their photostability, biocompatibility, low toxicity and low price [15,47,48,49]. The integration of excellent fluorescent properties of porphyrin macrocycles in CDs give raise to hybrid materials with outstanding optical properties to be exploited in both sensing and diagnostic fields.

On this matter, a Near-IR ratiometric fluorescence sensor has been proposed by Zao and coworkers: using corn bract as raw material and a solvothermal method, the researchers prepared a CD-based nanohybrid system for Hg^2+^ detection in serum and river water samples [50]. The obtained nanohybrid sensor exhibited dual fluorescence emission at 470 and 678 nm, which may originate from the intrinsic structure of CDs and chlorophyll-derived porphyrins, respectively. In the presence of Hg^2+^, the fluorescence at 678 nm could be remarkably quenched, while the fluorescence intensity at 470 nm was only slightly altered. The construction of this ratiometric sensor was easily achieved during the CDs preparation process, without post modification or mixing with other fluorophores: The procedure is simple, inexpensive, and environmentally friendly. The fluorescence intensity ratio, at 470 and 678 nm, exhibited a good linear relationship in the Hg^2+^ concentration range from 0 to 40 μM, with a detection limit of about 9.0 nM, making it potentially suitable for application in biomedicine study, environmental protection and food safety.

**Figure 8:**
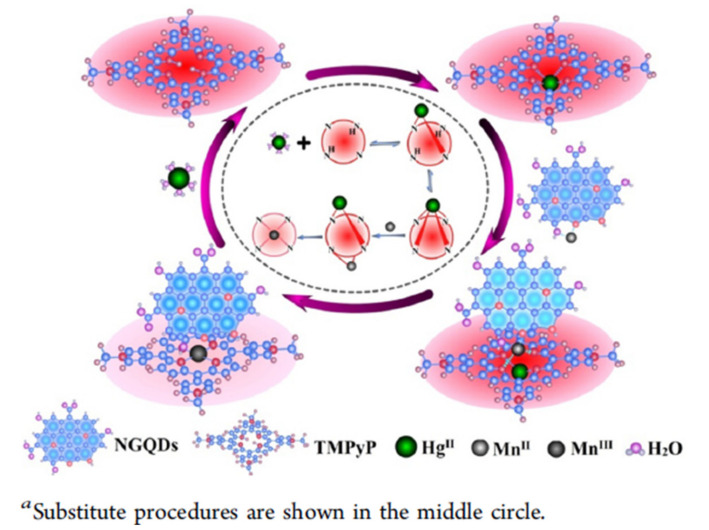
Schematic illustration of the synergistic effect of nitrogen-doped graphene quantum dots (NGQDs) and Hg^II^ in accelerating the coordination rate of Mn^II^ and TMPyP^a^. Adapted with permission from [51]. Copyright 2018, American Chemical Society.

Another sensitive, selective and multisignal method for determining traces of the most stable form of mercury pollution, Hg^2+^ has been proposed by Peng and coworkers [51]. They exploited a synergistic mechanism in which the relatively larger Hg^2+^ ions deform a porphyrin nucleus of TMPyP, which is favorable for attacking small divalent metal ions carried by nitrogen-doped graphene quantum dots (NGQDs) from the back (Figure 8). The NGQDs were synthesized with a two steps hydrothermal method as the authors described in a previous work [52]. The formation of metalloporphyrin was accompanied by the absorption red-shift and fluorescence quenching of TMPyP; simultaneously, the fluorescence of NGQDs is gradually enhanced because of the inner filter effect between porphyrins and NGQDs.

In detail, they observed that under the optimized reaction conditions (40 μM Mn^2+^, 20.0 μg L^−1^ NGQDs, and pH = 7.0), the TMPyP emission (λex = 420 nm) at 658 nm gradually decreased with the Hg^2+^ concentration in a range of 2–200 nM, while the NGQDs emission band centered at 490 nm gradually increases due to the weaker “primary” inner filter effect. Moreover, they highlighted how a colorimetric evolution also could be visually monitored under the 365 nm UV irradiation, when the bright pink emission of TMPyP was largely quenched upon addition of Hg^2+^ with different concentrations, and the fluorescence of NGQDs was gradually recovered with a bright blue emission appearing. Thus, the ratiometric fluorescence and colorimetric methods are particularly suitable for application in complex environmental and biological conditions.

A further facile method based on a one-pot hydrothermal approach using TPP or its Pd(II) or Pt(II) complex as a carbon precursor has been proposed by Fengshou’s group for the preparation of new N-rich metal-free CQDs or M-CQDs [53]. Similar to other CQDs reported in literature [54], the proposed CQDs and M-CQDs in aqueous solution exhibited strong luminescence. Typically, the oxygen functional groups on the surfaces of CQDs contribute not only to water solubility but also to their strong interaction with metal ions. The authors have studied the impact of different metal ions on the PL intensity of CQDs and M-CQDs and observed that the fluorescence intensities of CQDs were significantly decreased in the presence of Fe^3+^, while the other ions Ca^2+^, Hg^2+^, Na^+^, Mg^2+^, Mn^2+^, K^+^, Fe^2+^, Co^2+^, Ni^2+^, Al^3+^, Cu^2+^, Pb^2+^, and Zn^2+^ displayed weak or even negligible effects on their fluorescence intensities. Besides, the limit of detection for Fe^3+^ was figured out to be 3.7 mM. The high sensitivity together with the high selectivity for Fe^3+^ makes the CQDs a promising fluorescent sensing platform for the highly efficient detection of Fe^3+^.

**Figure 9:**
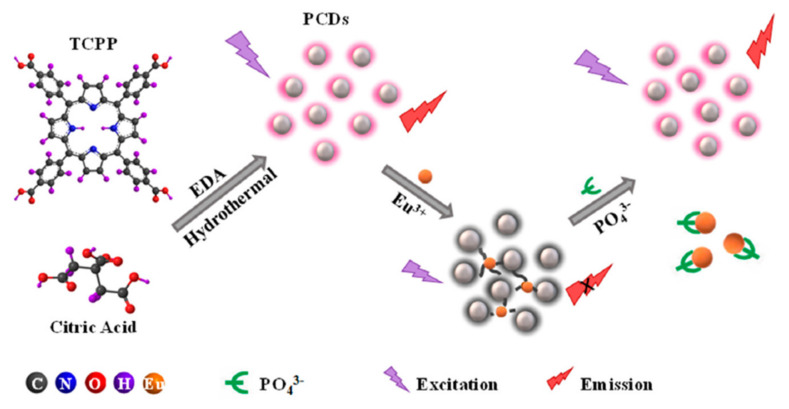
Schematic representation of the procedure for the preparation of porphyrin-based carbon dots (PCDs) and the sensing of phosphate. Adapted with permission from [55]. Copyright 2020, MDPI.

Always exploiting the excellent optical properties of CDs with the unique structure of TCPP, Wu and coworkers prepared a PCDs through a one-step hydrothermal approach in the presence of citric acid and ethanediamine as precursor [55]. By europium ion regulation, a simple phosphate detection “turn off-on” method was established, with PCDs as the fluorescence sensing probe (Figure 9). Phosphate plays pivotal roles both in living organisms, being essential constituent of nucleic acid, and in ecosystem as an indicator of organic pollution. Therefore, the quantitative determination of phosphate is of vital importance. The authors have demonstrated that the PCDs give rise to the optimal photoluminescence at λex/λem = 375/645 nm, that is turned off, via static quenching, when Eu^3+^ is added into PCDs, because the high affinity between the carboxylate groups, located on the surface of PCDs, and the lanthanide ion. On the other hand, when phosphate is introduced, Eu^3+^ shows a stronger affinity for phosphate than carboxylate groups. The addition of phosphate to PCDs-Eu^3+^ aggregates can recover the fluorescence intensity with a five-fold enhancement of this system, due do the desorption of Eu^3+^ from the surface of the PCDs, by utilizing the competition between the oxygen atom donors from the phosphate groups and those from the carboxylate groups on the surface of PCDs for Eu^3+^.

The authors have also studied the fluorescence response of PCDs-Eu^3+^ towards other cations and anions, and of the PCDs-Eu^3+^-PO_4_^3−^ system in the presence of a strong excess of interference groups, highlighting the selectivity and high sensitivity of the prepared system.

**Figure 10:**
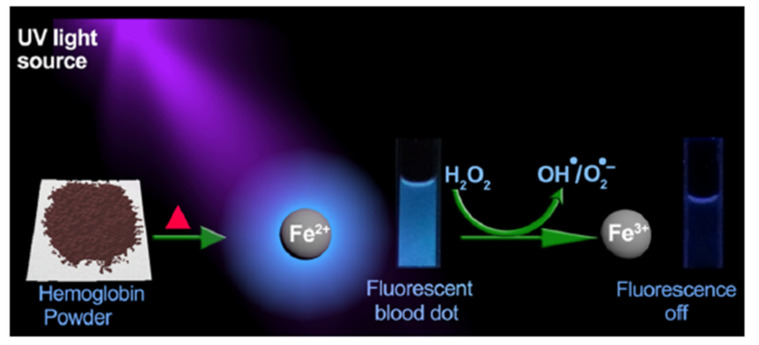
Pictorial representation of the synthesis and hydrogen peroxide sensing by a fluorescent blood dot. Adapted with permission from [56]. Copyright 2018, American Chemical Society.

Another species of remarkable importance in industry, food, medicine, biology, and clinical purposes is represented by the H_2_O_2_; in particular, an over production of this molecule is one of the major biochemical features of cancer cells. Hence, the recent increasing interest of the researchers to develop different techniques for H_2_O_2_ detection. In the presence of a suitable activator, the high content of hydrogen peroxide within cancer cells potentially can serve as a pro-drug by producing hydroxyl/superoxide radical, with cytotoxic properties through oxidative DNA damage. In view of these facts, Chakraborty and co-workers have synthesized Fe^2+^-containing carbon dots from hemoglobin, namely, BD, that can simultaneously act as a H_2_O_2_ sensor and a pro-drug activator [56]. Interestingly, the authors have observed that with a gradual increase in H_2_O_2_ concentration, the intrinsic fluorescence of BD at 405 nm gradually decreased because of the oxidation of the CDs surface hydroxyl groups, induced by the presence of the highly reactive radical species generated during the reaction between heme iron (Fe^2+^) and hydrogen peroxide (Figure 10). As a confirmation of this hypothesis, the same sensing experiment (reaction between BD (Fe^2+^) and H_2_O_2_) has been carried out in the presence of a radical scavenger, such a thiourea, and as expected, the fluorescence quenching of BD was prevented, indicating that this phenomenon is primarily caused by hydroxyl/superoxide radicals. On the other hand, they observed a fluorescence insensitivity testing two different carbon dots devoid of Fe^2+^ so to demonstrate that Fe^2+^ present in BD is mainly responsible for free radical generation during H_2_O_2_ sensing. Hence, the potential multitasking applications of this BD emerge, including biosensing, bioimaging and pro-drug activation for the selective killing of cancer cells.

**Figure 11:**
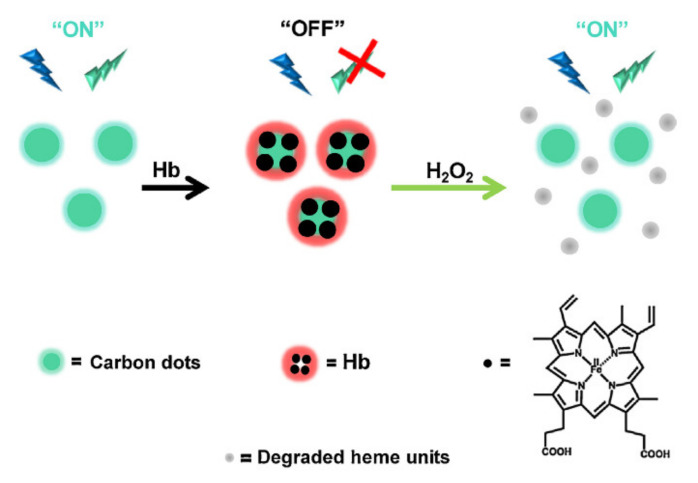
Proposed mechanism for H_2_O_2_ sensing via carbon dots’ (CDs) fluorescence “on–off–on”. Adapted with permission from [57]. Copyright 2018, Elsevier.

In the same way, Bhunia and coworkers presented a new fluorescence “on–off–on” sensing approach for the detection of hydrogen peroxide, using carbon dots prepared through a single-step carbonization procedure, using LPA as the carbon source [57]. Initially, the fluorescence of the LPA-CDs was quenched by addition of Hb, i.e., fluorescence “off” state, induced through π–π interaction between the heme units of Hb and the aromatic ring system of CDs. In particular, maximum quenching (~97%) was observed after incubation of the CDs with 50 μM Hb. However, H_2_O_2_ addition fully recovered the LPA-CDs fluorescence (fluorescence “on”); almost 100% fluorescence recovery was attained following addition of 100 mM H_2_O_2_ (Figure 11). The authors highlighted how the dramatic fluorescence recovery effect was likely due to degradation of hemoglobin combined with a unique radical scavenging mechanism affected by the LPA-CDs. The LPA-CDs H_2_O_2_ sensing scheme is simple, easy to perform, eco-friendly and exhibits good sensitivity.

## 5. Miscellaneous

**Figure 12:**
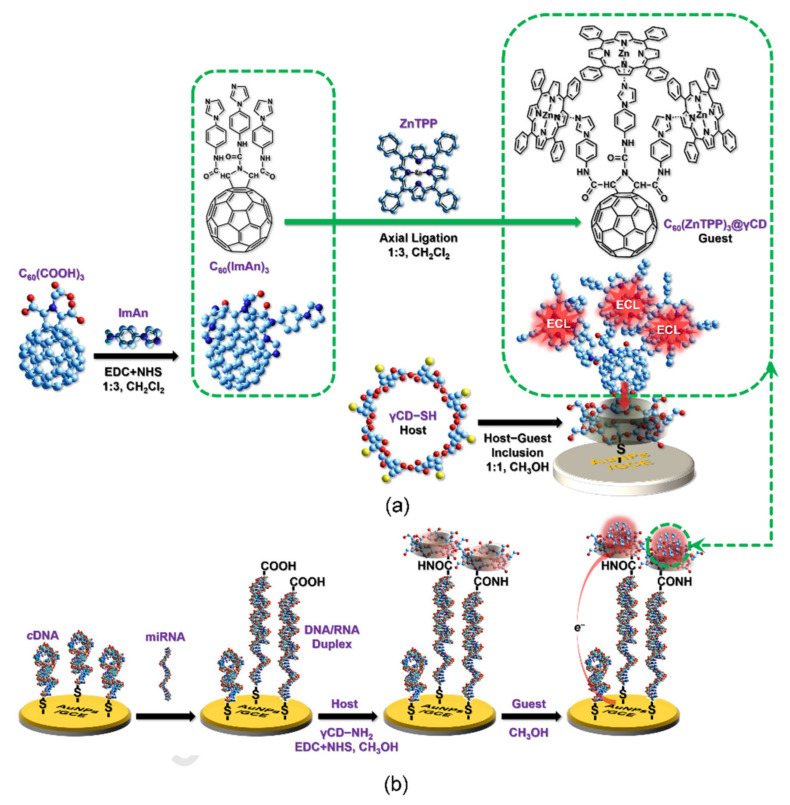
(**a**) Schematic illustration of the C_60_/(ZnTPP)_3_/γCD included in γCD-SH host, assembled on AuNPs. (**b**) Simplified miRNA assay developed by Deng and coworkers. Adapted with permission from [59]. Copyright 2020, Elsevier.

We conclude this survey illustrating few examples of sensors using porphyrins combined with other forms of carbon materials, which generally are less exploited in this field, like fullerene. In that regard, two sensor systems assembling this carbon allotropic form with porphyrin macrocycle have been recently reported in literature, both detecting molecules of biological interest. An electrochemical device was developed by Zhang and Li for the measurement of DA by immobilizing on GC electrode a system consisting of C_60_/porphyrin-diazocine-porphyrin dyad held together by non-covalent π–π interactions [58]. The electrochemical determination of DA was carried out by DPV in 0.2 M PBS, exploring the concentration range of 0–200 μM, following the well-resolved oxidation peak at 0.136 V. Good LOD value of 0.015 μM, satisfying repeatability and selectivity in the presence of common interfering species characterized the fabricated sensor. Deng and coworkers developed an elegant multicomponent system basing on host-guest chemistry of γ-CD, including fullerene and Zn porphyrins for the *sub*-picomolar detection of a model miRNA. Fabrication route and working mechanism are depicted in Figure 12 [59]. In this system, the hybrid C_60_(ZnTPP)_3_, was noncovalently but firmly embedded in the hollow of γCDs and anchored on AuNPs, functionalized with tiolated-γCDs that acted as hosting receptors for guest inclusion.

The obtained ensemble was uniform in both size and stoichiometry, so it was used for the in situ terminal labelling strategy during the recognition-induced allosteric event. The hybrid acted as ECL generator and afforded a neat signal enhancement following the detection of model miRNA marker. This work demonstrated the possibility to adapt macrocyclic chemistry for refined biotransducers and efficient ECL amplifiers with excellent potentials in bioassays.

As a final example, we present the hybrid system developed by Yimit et coworkers, who synthesized TCPP composited with graphitic carbon nitride g-C_3_N_4_ material through grinding method and ethanol dispersion and employed to OWG gas sensors by surface sensitization [60]. Under different pH conditions, the TCPP-g-C_3_N_4_ based sensor showed different behaviors and different optical properties depending on the aggregation extent, as confirmed by FESEM and UV–VIS studies. In particular, the authors found that the TCPP-g-C_3_N_4_ film under basic condition (pH = 11), where TCPP formed H-type aggregates on the film surface, had a good response to SO_2_ (0.1–100 ppm) and H_2_S (1–100 ppm), which can be explained by a protonation-deprotonation mechanism. Porphyrins in this case gave vermicular-like aggregated forms, with the g-C_3_N_4_ molecules existing in interstitial space and favoring gas adsorption.

## 6. Conclusions

The scientific community is incessantly engaged in discovering efficient technological solutions to apply in disparate fields dealing with health, safety, environment or communication. In this context, the production of high-performance chemical sensors able to rapidly and selectively detect chemical species of potential impact, as pollutants or incidentally released substances, is a more and more concrete imperative to fulfil, especially at this historic moment in which planet preservation, from water to air environments, cannot be taken for granted and postponed. These sensors require reliable and high-performance sensing materials, thus great efforts have to be channeled in producing ex novo materials or integrating those already existing to achieve hybrid nanostructures with amplified sensing efficiencies respect to the individual components. A plethora of combinations can be of course realized. Herein we report on the winning approach that uses porphyrins and CNMs entities to produce hybrid materials for the efficient detection of target analytes, both in liquid and gaseous phase. Carbon nanomaterials are indeed an excellent sensing platform, with robust intrinsic electronic and optical properties, conductivity and high surface area allowing for many simultaneous interactions with guest molecules. On the other hand, the richness of physicochemical properties and the chemical liability make porphyrins valuable sensing units easy to couple with different transducers.

The recent developments show that the more traditionally explored systems combining porphyrins with graphene or carbon nanotubes are still valid sensing systems, especially for the electrochemical detection of metal ions (Cd^2+^, Fe^3+^, Zn^2+^ [28,33,38]) or compounds of biological relevance in aqueous media [25,29,30,31,37]. Furthermore, novel configurations in FET devices using porphyrins/CNMs are increasingly used in chemical [43,44] and biological sensors fabrication, the latter commonly exploiting aptamer sensing strategy [35].

A quite emerging role is indeed the use of CDs for sensing purpose, not relegating their applications as good carriers and promising agents in nanomedicinal theranostics. Different forms of CDs prepared from a variety of carbon source can be used for the optical detection of ions of vital relevance, as Hg^2+^, Fe^3+^ and PO_4_^3−^ [50,51,53,55] or hydrogen peroxide, thanks to the use of Hemoglobin source to achieve the nanomaterial or combined in the hybrid, respectively [56,57].

These remarks suggest that chemical sensing with porphyrins/CNMs hybrid materials still offers many opportunities to be exploited for advancing in this field. Future efforts in the areas of synthesis, electronics and data analysis will surely result in novel sensor performances and applications in the upcoming years.

## Data Availability

No new data were created or analyzed in this study. Data sharing is not applicable to this article.

## Abbreviations

4-NP	4-nitrophenol
APTPP	5-(4-aminophenyl)-10,15,20-triphenylporphyrin
BD	blood-dots
BIA	bioelectrical impedance assay
BQ	benzoquinone
BTX	benzene-toluene-xylene
CC	catechol
CDs	carbon dots
CNMs	carbon nanomaterials
CNTs	carbon nanotubes
CNT-thread	carbon nanotube immobilized cellulose yarn
CQDs	carbon quantum dots
CrGO	chemical reduced graphene oxide
CS	chitosan
CTC	circulating tumor cell
CV	cyclic voltammetry
DA	dopamine
DPV	differential pulse voltammetry
ECL	electrochemiluminescence
ET	electron transfer
F-SWCNTs	pyridyl-functionalized single walled carbon nanotubes
FESEM	field emission scanning electron microscopy
FET	field-effect transistor
GAG	glycosaminoglycan
GCE	glassy carbon electrode
GO	graphene oxide
GQDs	graphene quantum dots
GrGO	green reduced graphene oxide
GSH	glutathione
GSs	graphene nanosheets
Hep	heparine
HP	hematoporphyrin
HQ	hydroquinone
ITO	indium tin oxide
LOD	limit of detection
LPA	L-penicillamine
M-CQDs	metal-doped carbon quantum dots
mi-RNA	micro-RNA
MWCNTs	multi-walled carbon nanotubes
NB	nitrobenezene
NDBA	*N*-nitrosodibuthylamine
NDEA	*N*-nitrosodiethylamine
NDMA	*N*-nitrosodimethylamine
NGQDs	nitrogen-doped graphene quantum dots
NIR	near infra-red
NPs	nanoparticles
NS	nanospheres
OEP	octaethylporphyrin
OWG	optical waveguide
PBS	phosphate-buffered saline
PCDs	porphyin-based carbon dots
PEC	photoelectrochemical
PL	photoluminescence
PLSR	partial-least-square regression
PSF	porous silk-fibroin
QMBs	quartz microbalances
rGO	reduced graphene oxide
SPCE	screen printed carbon electrode
SWNTs	single walled nanotubes
TAPP	5,10,15,20-tetrakis(4-aminophenyl)porphyrin
TCPP	5,10,15,20-tetrakis(4-carboxyphenyl)porphyrin
TFPP	5,10,15,20-tetrakis(pentafluorophenyl)porphyrin
TMB	3,5,5-tetramethylbenzidine
TMPP	5,10,15,20-tetrakis(4-methoxyphenyl)porphyrin
TMPyP	5,10,15,20-tetrakis(1-methyl-4-pyridinio)porphyrin tetra(*p*-toluenesulfonate)
TOAB	tetraoctylammonium bromide
TOBPP	5,10,15,20-tetrakis(4-butyloxyphenyl)phenylporphyrin
TPP	5,10,15,20-tetraphenylporphyrin
TPyP	5,10,15,20-tetrakis(4-pyridyl)porphyrin
Trp	tryptophan
VOC	volatile organic compounds
γ-CD	γ-ciclodextrines
